# Surfactant-Induced Reconfiguration of Urea-Formaldehyde Resins Enables Improved Surface Properties and Gluability of Bamboo

**DOI:** 10.3390/polym13203542

**Published:** 2021-10-14

**Authors:** Lulu Liang, Yu Zheng, Yitian Wu, Jin Yang, Jiajie Wang, Yingjie Tao, Lanze Li, Chaoliang Ma, Yajun Pang, Hao Chen, Hongwei Yu, Zhehong Shen

**Affiliations:** College of Chemistry and Materials Engineering, Zhejiang Provincial Collaborative Innovation Center for Bamboo Resources and High-Efficiency Utilization, National Engineering and Technology Research Center of Wood-Based Resources Comprehensive Utilization, Key Laboratory of Wood Science and Technology of Zhejiang Province, Zhejiang A&F University, Hangzhou 311300, China; m13070173339@163.com (L.L.); z541962382@icloud.com (Y.Z.); Y1tianWu@163.com (Y.W.); Yangjin_0127@163.com (J.Y.); laowangwjj0126@163.com (J.W.); taoyingjie123@126.com (Y.T.); lilanze112@163.com (L.L.); qq843543030@163.com (C.M.); haochen10@fudan.edu.cn (H.C.)

**Keywords:** surfactant, urea-formaldehyde resin, bamboo, laminated bamboo veneer, gluability

## Abstract

The high-efficiency development and utilization of bamboo resources can greatly alleviate the current shortage of wood and promote the neutralization of CO_2_. However, the wide application of bamboo-derived products is largely limited by their unideal surface properties with adhesive as well as poor gluability. Herein, a facile strategy using the surfactant-induced reconfiguration of urea-formaldehyde (UF) resins was proposed to enhance the interface with bamboo and significantly improve its gluability. Specifically, through the coupling of a variety of surfactants, the viscosity and surface tension of the UF resins were properly regulated. Therefore, the resultant surfactant reconfigured UF resin showed much-improved wettability and spreading performance to the surface of both bamboo green and bamboo yellow. Specifically, the contact angle (CA) values of the bamboo green and bamboo yellow decreased from 79.6° to 30.5° and from 57.5° to 28.2°, respectively, with the corresponding resin spreading area increasing from 0.2 mm^2^ to 7.6 mm^2^ and from 0.1 mm^2^ to 5.6 mm^2^. Moreover, our reconfigured UF resin can reduce the amount of glue spread applied to bond the laminated commercial bamboo veneer products to 60 g m^−2^, while the products prepared by the initial UF resin are unable to meet the requirements of the test standard, suggesting that this facile method is an effective way to decrease the application of petroleum-based resins and production costs. More broadly, this surfactant reconfigured strategy can also be performed to regulate the wettability between UF resin and other materials (such as polypropylene board and tinplate), expanding the application fields of UF resin.

## 1. Introduction

As a natural organic material as well as a sustainable resource, wood has been widely relied on and developed for a long period all over the world due to its facile processing, light weight, good elasticity, impact resistance, low density, rich mesoporous structure, and other characteristics [1,2]. However, the supply of available wood is far from meeting the world’s needs. Therefore, we urgently need to explore effective alternative materials [3]. Bamboo is a natural biomass composite with not only renewable, biodegradable, and carbon sequestration but also an impressive faster growth rate than wood, a high annual regeneration rate after harvesting, and excellent mechanical properties [4,5]. It is thus considered a promising ingredient substitute for wood.

Bamboo is hollow inside, and tubular parts are formed between the nodes, which can effectively resist the bending force [6]. However, the connection and bonding of circular cross-sections are difficult, and the pipes cannot be used in applications requiring flat surfaces [5]. In addition, the thickness of the stalk wall gradually becomes thinner from the base to the top of the stalk, and the bamboo fiber content is not uniformly distributed with the change of the wall thickness. These changes in geometric and mechanical properties also limit the structure of whole bamboo [7]. At present, in order to avoid the defects caused by the natural shape of bamboo, there have been developments of bamboo-based composite materials using industrialized production processes, including, for example, bamboo plywood, laminated bamboo lumber, and bamboo scrimber. They are widely used in furniture, flooring, construction, civil engineering, and other fields [8,9,10]. However, due to the absence of transverse ray cells in bamboo tissues and the unobvious flow, wetting, and penetration depth of the liquid adhesive on the surface of the bamboo, the crosslinking of the adhesive and the bamboo material is unable to achieve the mechanical coupling effect with the wood [11,12,13]. In addition, bamboo green and bamboo yellow contain a certain amount of hydrophobic substances, including wax, fat, SiO_2_, bamboo film, and others, which will negatively affect the wettability and adhesion of bamboo [14]. As a result, the current bamboo production generally removes bamboo green and bamboo yellow, which leads to the waste of bamboo raw materials and a low utilization rate [15]. Therefore, improving the wettability and gluability properties of the adhesives in bamboo materials is of great significance to reduce the production costs of bamboo products and accelerate the development of the bamboo industry.

However, most of the research on wood and bamboo is focused on the surface engineering of the material, such as the introduction of coating and reasonable design [16,17]. Moreover, the research on enhancing the gluability properties of bamboo mostly focuses on the performance of the bamboo materials itself and rarely involves the regulation of the properties of the adhesives [18,19,20,21,22,23]. Currently, the main commonly used adhesives include amino, phenolic, and isocyanate, among which 95% of the adhesives are formaldehyde-based, with urea-formaldehyde (UF) as the most predominant [24]. Specifically, although there are some shortcomings, such as easy aging, poor water resistance, poor wettability, and the release of volatile organic compounds (VOCs) [25,26,27], the UF resin is a thermosetting resin formed by the condensation of urea and formaldehyde [28] that has been used in the manufacture of particleboard, medium-density fiberboard, and indoor plywood due to the characteristics of cheap and easy-to-obtain raw materials, a simple synthesis process, low curing temperature, short pressing time, wide curing conditions, colorless glue line, and good panel performance [29,30,31,32]. Therefore, in this present study, taking urea-formaldehyde (UF) resin as an example, the significant role of regulating the adhesive on the wettability and bonding performance is systematically studied. In a detailed manner, the surfactants that can reduce surface tension, increase solubility, and have the ability to improve wettability [33] are applied as modifiers to reconfigure UF resin, including three typical different types of surfactants (cetyltrimethylammonium bromide (CTAB) as an anionic surfactant, sodium dodecyl sulfate (SDS) as a cationic surfactant, and Span 80 as non-ionic surfactant). Impressively, the resultant surfactant reconfigured UF resin with regulated viscosity and surface tension can significantly improve its wettability and spreading performance to the surface of both bamboo green and bamboo yellow. When the optimized reconfigured UF resin is further applied for commercial bamboo veneer, the thus-formed laminated bamboo veneer product can also exhibit outstanding wettability, and the average peeling length is largely reduced during the dipping peel test. Moreover, our reconfigured UF resin can reduce the amount of glue spread to 60 g m^−2^, while the products prepared by the initial UF resin are unable to meet the requirements of the test standard, suggesting that this surfactant-induced reconfiguration provides an effective way to decrease the application of petroleum-based resins and reduce production costs. In addition, this surfactant reconfigured strategy has also shown positive effects in improving the wettability between UF resin and other materials, such as polypropylene (PP) board and tinplate, supplementing a reference for expanding the application fields of UF resin.

## 2. Materials and Methods

### 2.1. Materials

**Figure 1:**
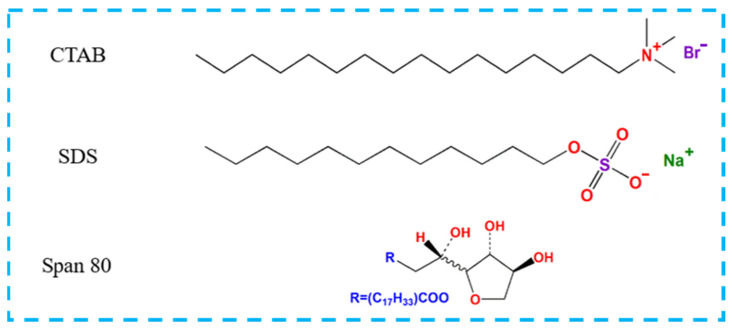
Molecular structure of surfactants.

Formaldehyde aqueous solution (HCHO, 37%) was obtained from Shanghai Zhanyun Chemical Co., Ltd., (Shanghai, China). Urea (CH_4_N_2_O, ≥99.0%) was obtained from Shanghai Lingfeng Chemical Reagent Co., Ltd., (Shanghai, China). Formic acid (HCOOH, 98%) was purchased from Guangdong Guanghua Sci-Tech Co., Ltd., (Guangdong, China). Sodium hydroxide (NaOH, 95%), cetyltrimethylammonium bromide (CTBA, AR), sodium dodecyl sulfate (SDS, AR), and Span 80 (SP) were purchased from Aladdin Industrial Corporation (Shanghai, China). All chemicals were used without further purification. Polypropylene (PP) boards were purchased from Guangzhou Chuangxin Rubber and Plastic Products Co., Ltd. (Guangzhou, China). Tinplates were purchased from Shenzhen Xinrong Stainless Steel Material Co., Ltd. (Shenzhen, China). Bamboo splits and bamboo veneers (produced by sawing the laminated bamboo lumbers into thin sheets) were kindly donated by Zhejiang Zhuangyi Furniture Co., Ltd. (Hangzhou, China). The molecular structures of all surfactants employed are listed in Figure 1.

### 2.2. Synthesis of Urea-Formaldehyde (UF) Resin and Reconfigured UF Resins

Urea-formaldehyde (UF) resin was prepared through the traditional “alkali-acid-alkali” three-step process. In detail, 264.6 g of formaldehyde solution was stirred in a four-neck reactor, and then the pH was adjusted to 8.0 with a 30% NaOH aqueous solution. Next, 95.14 g of urea was added into the formaldehyde solution for methylation reaction. The mixture was further heated to 90 °C and maintained at the temperature for 45 min. Next, under acidic conditions (pH = 5–5.5, adjusted with 30% HCOOH), the polycondensation reaction was carried out, and 18.98 g of urea was added until the reactant reached the target viscosity. When the temperature of the solution cooled to 70 °C, 6 g of urea was added to the reactor again. When the temperature of the solution in the reactor continued to cool to 40 °C, the pH of the solution was adjusted to alkaline conditions again (pH = 8.0, adjusted with 30% NaOH. As a result, the UF resin was thus obtained, which can be used after cooling to room temperature. The surfactant reconfigured UF resin was constructed by mixing UF resin with different amounts of CTAB at room temperature, including, for example, 0.5%, 1%, 1.5%, 2%, 2.5%, and 3%, which were based on the theoretical solid content of UF resin [34]. The resulting reconfigured UF resin was labeled x C-UF (x stands for CTAB content, C stands for CTAB). Similarly, S-UF resin and Sp-UF resin were SDS and Span 80 reconfigured UF resins, respectively.

### 2.3. Preparation of the Basal Substrates

The bamboo green and bamboo yellow used for contact angle measurement with the size of 50 mm × 3 mm × 10 mm (longitudinal × radial × tangential) were obtained from the bamboo splits without removing the bamboo green and bamboo yellow, respectively. The size of the bamboo veneer used for the dipping peel test was 75 mm × 75 mm × 3 mm. In addition, PP board, tinplate, and bamboo veneer were cut into a size of 40 mm × 40 mm × 10 mm for contact angle measurement.

### 2.4. Viscosity and Surface Tension Test

The viscosity of the resin was measured by NDJ-1 type rotational viscometer (Shanghai Lichen Bangxi Instrument Technology Co., Ltd., Shanghai, China) at a temperature of 25 ± 2 °C. The surface tension of the resin was tested with a contact angle measuring instrument (JC2000D1, Shanghai Zhongchen Digital Technology Instrument Co., Ltd., Shanghai, China) using the pendant drop method. Each sample was measured at 6 points, and the results were averaged.

### 2.5. Spreading Performance Test

A drop of different resin was dropped on the surface of the corresponding substrate, and then the recording of the contact angle (CA) images and optical photos of the resin and the substrate surface took place for 1 s and 60 s. After that, ImageJ software was used to measure the exact area of the resin before and after spreading on the substrate surface. By comparing the area value, the spreading performance of different resins on the surface of the corresponding substrate was studied. Each test was repeated 3 times.

### 2.6. Laminated Bamboo Veneers and Its Dipping Peel Test

Two bamboo veneers (75 mm × 75 mm × 3 mm) were bonded and formed laminated bamboo veneer using UF or 2.5% Sp-UF resin. The resin was manually applied to the surface of a bamboo veneer using the glue spread of 80 g m^−2^ and 60 g m^−2^, respectively. Subsequently, the laminated bamboo veneers were hot-pressed for 10 min at 110 °C and with 3.8 MPa of pressing pressure. All the panels were stored under an ambient condition for 24 h prior to testing. The dipping peel analysis follows the Chinese National Standard (GB/T 20240-2017) in which the specimens were submerged in 63 ± 3 °C water bath for 3 h and then dried at 63 ± 3 °C for 10 h. Finally, the specimens were taken out to observe the degree of peeling of the glue lines. Each test has 6 duplicate test pieces.

### 2.7. Characterization

The CA of resin at substrate was analyzed at room temperature using a contact angle meter (JC2000D1, Shanghai Zhongchen Digital Technic Apparatus Co., Ltd., Shanghai, China), where the specific CA result was an average value recorded from tests performed at six locations on the same sample surface. The morphology was observed by scanning electron microscopy (SEM, TM3030, Hitachi, Tokyo, Japan). The surface composition of the product was identified by X-ray photoelectron spectroscopy (XPS, ESCALAB 250XI, Thermo Fisher Scientific, Waltham, America) in which the binding energy was calibrated using the C 1s reference peak at 284.8 eV. An optical microscopy image of the bamboo veneer surface was taken using microscopy (THMS600, Nikon, Tokyo, Japan).

## 3. Results

### 3.1. The Effect of the Surfactants on the Viscosity and Surface Tension of the UF Resins

**Figure 2:**
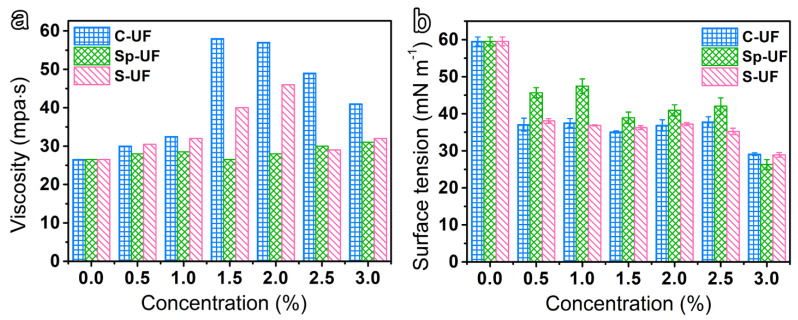
(**a**) Viscosity, and (**b**) surface tension of the UF and reconfigured UF resins.

Prior to applying the UF and reconfigured UF to the surface of the bamboo green and bamboo yellow, the viscosity and surface tension, these two significant characteristics of resin, were systematically investigated. It has been well-demonstrated that the viscosity of the resin will significantly affect its fluidity and permeability on the substrate [35]. As observed in Figure 2a, when CTAB and SDS are used to reconfigure the UF, the changes of their viscosity present a similar phenomenon; that is, the viscosity increases initially with the increase in the amount of surfactant and then reaches the maximum value, and, as the amount continues to increase, the viscosity will decrease instead. This is due to the fact that, for reconstructed UF resins containing such CTAB or SDS ionic-based surfactants, the increase in the ionic strength of the solution will increase the viscosity of the resin correspondingly, which will reach the maximum value when the micelles are formed in the solution [36]. However, when the proportion of surfactants continues to increase, there will be a large number of micelles in the solution, which repels the surfactant in the interfacial film, which will affect the accumulation mode and regularity of the surfactants and eventually lead to a corresponding weakening of the inhibitory force of particle movement, thus resulting in the decrease of viscosity [37]. To differentiate an ionic surfactant with a non-ionic surfactant, Span 80 has no electrostatic interaction with polymers. Therefore, the viscosity of the UF and Sp-UF shows negligible changes [38].

Surface tension is an important factor in evaluating the wettability potential of resin on the substrate [39]. In general, the smaller the surface tension of the solution, the better its wetting performance. Figure 2b shows the surface tension of the different UF resins. Typically, the changing trend of the surface tension of the surfactants in the solution has three forms: (1) For most inorganic electrolytes, the surface tension of the solution increases slowly with the increase of the solute concentration. (2) The surface tension of the solution slowly decreases with the increase of the solute concentration, such as some low-molecular-weight polar organics with weak hydrophilicity. (3) For amphiphilic organic compounds containing more than eight carbon atoms, the surface tension of the solution can be significantly reduced at low concentrations, and, when the concentration increases to a certain value, the surface tension of the solution does not decrease or decreases slowly [40]. Thus, based on the molecular structure of the three surfactants, Span 80 belongs to the substance described by the second type, while CTAB and SDS belong to the substance described by the third type. As expected, it can be observed from Figure 2b that all the types conform to the corresponding rules.

### 3.2. The Wettability of UF and Reconfigured UF Resins on the Bamboo Green or Bamboo Yellow Surface

**Figure 3:**
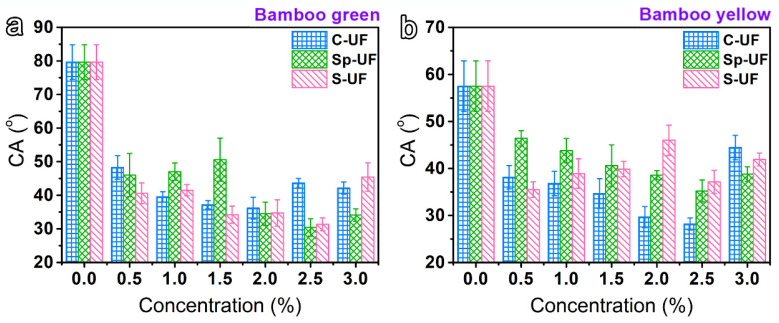
The CA curves of the UF and reconfigured UF resins on the surface of (**a**) bamboo green and (**b**) bamboo yellow.

Considering the regulated viscosity and surface tension of the resin after the surfactant reconstruction, we further recorded the CA value of the resin on the surface of the bamboo green and bamboo yellow, respectively, to directly compare the wettability of the various UF resins on the bamboo substrate. The CA value of the UF resin on the bamboo green and bamboo yellow without any surfactant is as high as ~79.6° and 57.5°, while, impressively, all three of the reconfigured UF resins exhibit much lower CA values than that of the UF resin (Figure 3). This implies that a much better wetting behavior between the resin and the bamboo surface could be realized with the surfactant-induced reconfiguration. Among them, it is evident that the surface wettability of bamboo yellow is better than that of bamboo green, which can be attributed to more polar groups contained on the surface of bamboo yellow [41]. Furthermore, considering the wettability of various resins on the surface of the bamboo green and bamboo yellow, the 2.5% Sp-UF (CA value of 30.5°) and 2.5% C-UF (CA value of 28.2°) is the most suitable for coating on the surface of the bamboo green and bamboo yellow, respectively.

### 3.3. Spreading Performance of the UF and Reconfigured UF Resins

**Figure 4:**
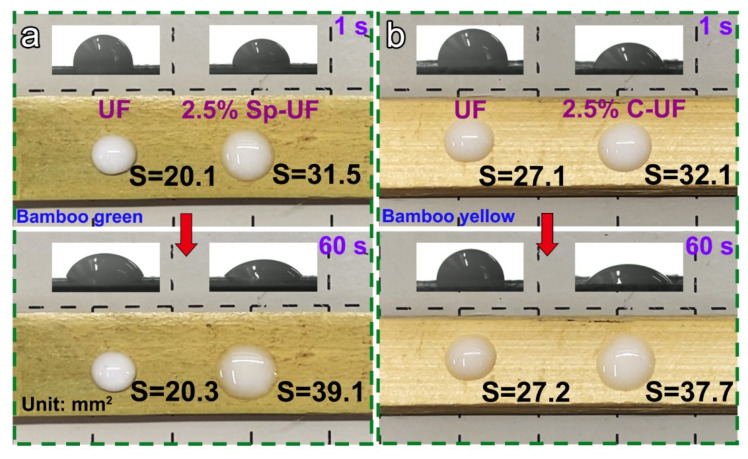
Optical photos and corresponding CA images of UF and reconfigured UF resins in contact with the surface of (**a**) bamboo green and (**b**) bamboo yellow for 1 s and 60 s.

As another factor affecting the adhesion performance, the diffusion ability of the UF, 2.5% Sp-UF, and 2.5% C-UF on the substrate was evaluated according to the results of the CA measurement. As shown in Figure 4, the recorded area changes over time when the UF and reconfigured UF resins are in contact with the same substrate. In detail, by comparing the optical photos and CA images of the UF and 2.5% Sp-UF resins dipped on the one bamboo green for 1 s, respectively, the area of a drop of 2.5% Sp-UF resin through software measurement can reach 31.5 mm^2^, while the area of a drop of the UF resin was only 20.1 mm^2^. More impressively, within 60 s, the spreading area of 2.5% Sp-UF resin on the bamboo green surface increased by 7.6 mm^2^, while the spreading area of the UF resin exhibited no observable change. Similarly, the spreading area of 2.5% C-UF resin on the bamboo yellow increased from 32.1 to 37.7 mm^2^ after waiting for 60 s; conversely, the spreading area of UF resin on bamboo yellow did not differ much. These results verify that the reconfigured UF resins possess a faster diffusion rate and stronger spreading ability on the bamboo substrates, which is consistent with the results in Figure 3. Therefore, the addition of surfactants can greatly enhance the spreading ability of UF resin on both bamboo green and bamboo yellow.

### 3.4. Morphology and Compositions of UF and Reconfigured UF Resins

**Figure 5:**
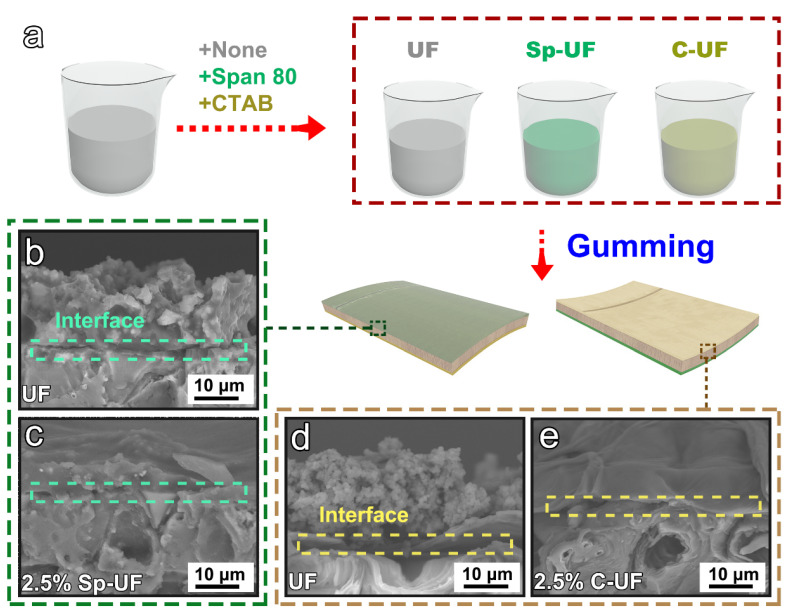
(**a**) Schematic procedure of the fabrication process. SEM images of (**b**) cured UF resin coated on the bamboo green, (**c**) cured 2.5% Sp-UF resin coated on the bamboo green, (**d**) cured UF resin coated on the bamboo yellow, and (**e**) 2.5% C-UF resin coated on the bamboo yellow.

Considering the excellent wettability and spreading performance of our reconfigured UF resins, we further uniformly coated the corresponding three resins on the surface of the bamboo green and bamboo yellow, respectively, as illustrated in the schematic diagram of the production process (Figure 5a). The morphology of the UF and corresponding reconfigured UF resin cured on the surface of the bamboo green and bamboo yellow was first characterized by a scanning electron microscope (SEM), as shown in Figure 5b,c. Regarding the bamboo green substrate, the cured UF resin is granular on the surface, and the cured 2.5% Sp-UF resin is evenly distributed on the surface to form a thinner film, indicating that the coupling of Span 80 improves the spreading performance of the UF resin on the bamboo green surface. Figure 5d,e shows that the 2.5% C-UF resin exhibits the same effect for the surface of bamboo yellow. The UF resin solution not only cannot be uniformly distributed on the surface of bamboo yellow but is also difficult to penetrate the inside, resulting in the cured UF resin on the surface granular. However, the reconfigured resin solution can almost completely penetrate the inside of the bamboo substrate instead.

**Figure 6:**
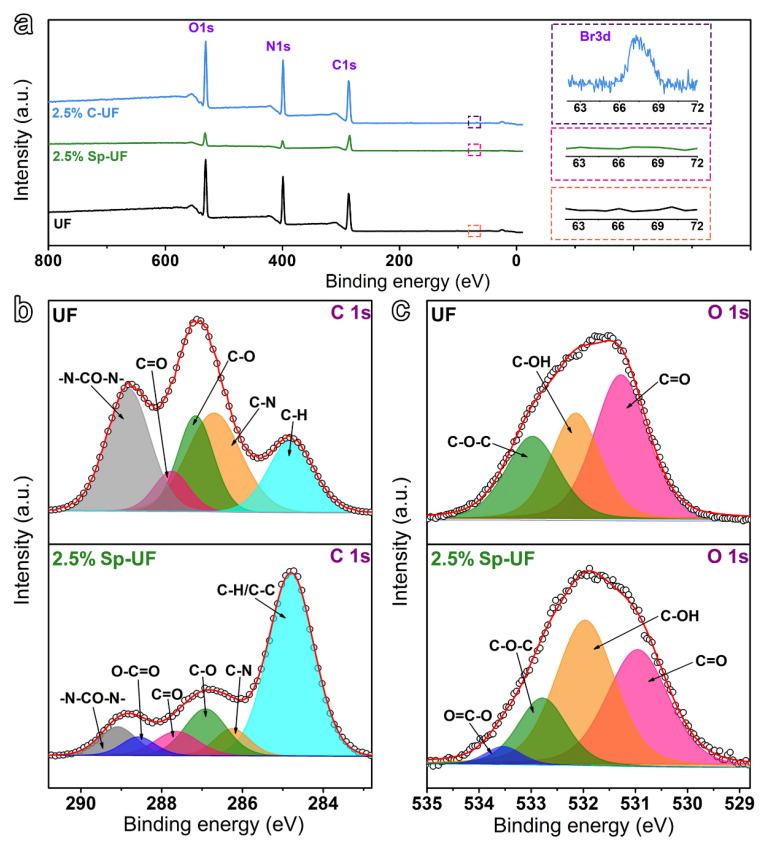
(**a**) XPS full spectra of the cured UF, 2.5% Sp-UF, and 2.5% C-UF resins. High-resolution (**b**) C 1s, and (**c**) O 1s XPS spectra of the UF and 2.5% Sp-UF resins.

The detailed compositions of the cured UF, 2.5% Sp-UF, and 2.5% C-UF resins were further investigated by X-ray photoelectron spectroscopy (XPS) spectrum. The full XPS survey spectra show the main components of the UF and two reconfigured UF resins are C, N, and O, which is also consistent with their structure (Figure 6a). Notably, unlike the others, an additional Br 3d is detected in the 2.5% C-UF resin due to its special molecular structure (see Figure 1), further suggesting that CTAB has been successfully coupled with the UF resin evenly. In addition, by comparing the C 1s of UF and 2.5% Sp-UF resin shown in Figure 6b, the high-resolution spectrum of the UF resin can be resolved into five peaks at 284.8, 286.7, 287.2, 287.8, and 288.8 eV, corresponding to the C−H, C−N, C−O, C=O, and −N−CO−N− components. In contrast, with the coupling of the Span 80 surfactant, there is a significant increase of the peak at 284.8 and a new peak at 288.6, which can be identified as the signals of the C−H and O−C=O groups of the Span 80, respectively [42,43,44]. This demonstrates that Span 80 is coupled with the UF resin. Moreover, regarding the high-resolution O 1s spectrum, the presence of the O−C=O group belonging to Sp 80 is also illustrated by the fitting result (Figure 6c) [45,46].

The above results prove that the surfactant-coupled method we proposed can successfully realize the reconfiguration of the UF resin, thus regulating the viscosity and surface tension. The reconfigured UF resin overcame the problem of poor wettability and spreading performance on the surface of both the bamboo green and bamboo yellow, thereby obtaining a much-improved contact interface between the resin with the bamboo materials substrate. Therefore, it is believed that the reconfigured resins can be applied to improve the bonding performance between bamboo veneers, which are currently widely used as bamboo-based decoration and construction material in practical applications [47].

### 3.5. The Applications of Modified UF Resins on Laminated Bamboo Veneers

**Figure 7:**
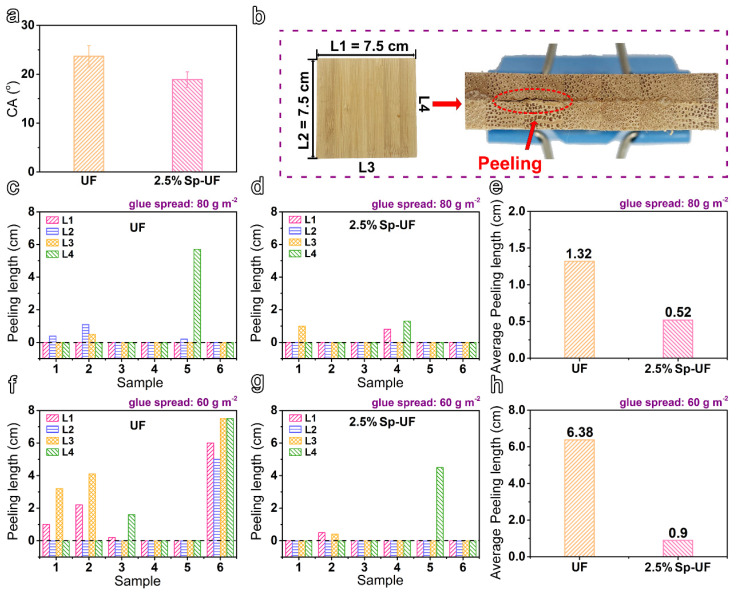
(**a**) The CA values curve of UF and 2.5% Sp-UF resins on the bamboo veneer surface, (**b**) Optical photos of the laminated bamboo veneer and its peeling of the glue line, (**c**,**d**) Peeling length graphs of UF and 2.5% Sp-UF resins and (**e**) the comparison graph of their average peeling length at glue spread of 80 g m^−2^, (**f**,**g**) Peeling length graphs of UF and 2.5% Sp-UF resins and (**h**) the comparison graph of average peeling length at glue spread of 60 g m^−2^.

According to the comprehensive performance of various reconfigured UF resins, the UF and 2.5% Sp-UF resin were, therefore, selected to be applied to the laminated bamboo veneer. The resultant wettability and bonding performance are shown in Figure 7. Obviously, a lower CA value of 2.5% Sp-UF resin coated on the laminated bamboo veneer is realized as expected, implying enhanced wettability (Figure 7a). Figure 7b displays the optical photos of the bamboo veneer and its peeling of the glue line, where the four sides of the bamboo veneer are named L1, L2, L3, and L4, and the length of each side is 7.5 cm. It is worth pointing out that the above parameters are all selected according to the requirements of the test standard. We then performed the dipping peel test after the bamboo veneer was glued; a peeling length was thus obtained when the glue line on one side of the glue-laminated bamboo veneers fell off, and this was the basis for evaluating the adhesion performance of the resin. Specifically, we investigated the dipping peel test of glue-laminated bamboo veneers containing UF and 2.5% Sp-UF resin with glue spreads of 80 g m^−2^ and 60 g m^−2^, respectively. The peeling length of each sample of six laminated bamboo veneers prepared with the UF or 2.5% Sp-UF resin under the glue spread of 80 g m^−2^ was recorded as presented in Figure 7c,d. Impressively, in comparison with the average peeling length of the UF resin, a peeling length of only ~0.52 cm of 2.5% Sp-UF was achieved, which is much lower than that of the UF resin without reconfiguration (Figure 7e). Moreover, a similar positive effect was also shown when the glue spread was decreased to 60 g m^−2^, where the average peeling length was reduced from 6.38 cm to 0.9 cm (Figure 7f–h). In addition, it is worth pointing out that, according to the Chinese National Standards (GB/T 20240-2017), the total stripping length of at least five of the six specimens is less than 1/3 of the full length of the glue layer, so it can be determined that the products are in compliance with the standard requirement. Therefore, these results demonstrate our reconfigured UF resin can reduce the amount of glue spread to 60 g m^−2^, while the products prepared by the initial UF resin are unable to meet the standard. In addition, by comparing the results with the previously reported literature, the amount of adhesive used in this study is much lower than the previous amounts (including 200 g m^−2^, 220 g m^−2^, and 250 g m^−2^) [48,49,50], together with maintaining excellent gluability performance. These aspects prove that our method provides a way to reduce production costs.

**Figure 8:**
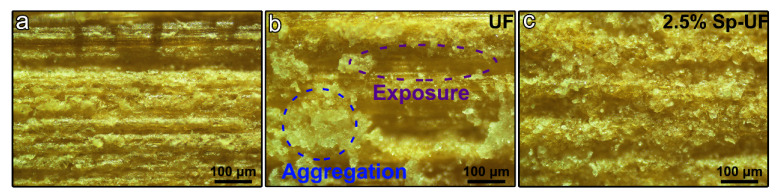
Optical microscopy images of (**a**) unglued bamboo veneer and glued bamboo veneers with cured (**b**) UF resin and (**c**) 2.5% Sp-UF resin at the glue spread of 60 g m^−2^.

To investigate the failure of the cured UF and 2.5% Sp-UF resins under the glue spread of 60 g m^−2^ in depth, optical microscopy images of the unglued bamboo veneer and glued bamboo veneers were observed (Figure 8). It can be seen that the UF resin is unevenly distributed on the surface of the bamboo veneer, resulting in a lack of adhesive in some places, while the 2.5% Sp-UF resin is evenly distributed and almost completely covers the surface. This phenomenon reveals that the interfacial properties of the resin and the bamboo surface, including the wettability and spreading properties, will greatly affect the distribution of the resin during the gluing process, thereby affecting the gluability performance. These results further prove that the surfactant-induced reconfigured resin can improve its wettability to the bamboo laminate, thus making it exhibit higher adhesion performance than the UF resin and hold great potential in promoting the comprehensive and efficient utilization of bamboo-based resources.

### 3.6. The Wettability Study of UF and Reconfigured UF Resins on the Other Substrates

**Figure 9:**
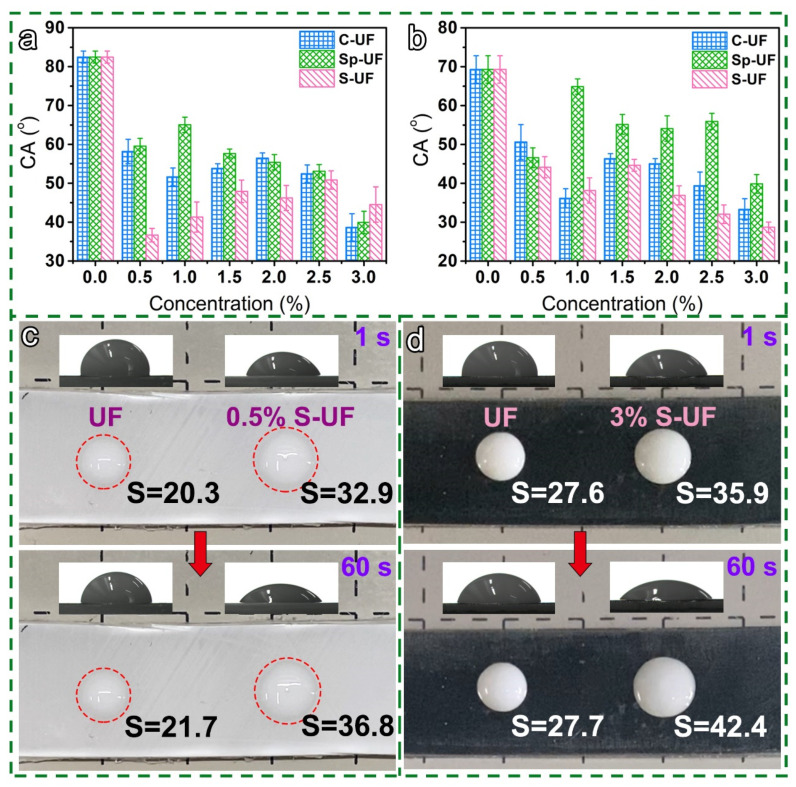
The CA curves of the UF and reconfigured UF resins on the surface of (**a**) PP board, and (**b**) tinplate. Optical photos and corresponding CA images of UF and reconfigured UF resins in contact with the surface of (**c**) PP board and (**d**) tinplate for 1 s and 60 s.

In addition to the outstanding performance on the bamboo substrate, we also verified the application of our designed UF resin on other substrates, including PP board and tinplate. The corresponding CA results of various resins show that, although the regulation of wettability on the different substrates is inconsistent, for example, the S-UF possesses the smallest CA value on the PP board, this surfactant-induced reconfiguration can also indeed improve the wettability of UF resin on other substrates (Figure 9a,b). Additionally, we also compared the spreadability of the optimized reconfigured UF resins on the two substrates (Figure 9c,d). The resultant spreading area of 0.5% S-UF resin on the PP board and tinplate increased from 32.9 to 36.8 mm^2^ and from 35.9 to 42.4%, respectively, which undoubtedly shows a faster spreading speed and stronger spreading ability than the UF resin. In short, the induction of the surfactants can also improve the wettability and spreadability of the UF resin to PP boards and tinplate, which provides a reference for expanding the application fields of UF resin.

## 4. Conclusions

In summary, the wettability of urea-formaldehyde resin reconfigured with three surfactants (CTAB, Span 80, and SDS) on the series of bamboo substrates (bamboo green, bamboo yellow, and bamboo veneer) has been greatly improved. Specifically, the CA values of the reconfigured UF resins on the different substrates have been obviously reduced. Among them, the CA value of the optimized Span 80 reconfigured UF resin on the surface of the bamboo green decreased from 79.6° to 30.5°. Meanwhile, after adding the appropriate amount of CTAB, the CA value of the UF resin on the surface of the bamboo yellow decreased from 57.5° to 28.2°. Therefore, according to the comprehensive performance of the reconfigured UF resins, the 2.5% Sp-UF resin is selected to be applied to the laminated bamboo veneer. The final experimental results showed that the laminated bamboo veneers prepared with a lower glue spread (60 g m^−2^) of 2.5% Sp-UF resin can still meet the requirement determined by the Chinese National Standard (GB/T 20240-2017), which proves that the research in this paper could provide an efficient way to cut down the production costs and reduce the application of the petrochemical-based resin. Furthermore, this facile strategy can also be extended to other materials, such as polypropylene plates and tinplate.

## Data Availability

Not applicable.
